# Strategies for Mitigating Dissolution of Solid Electrolyte Interphases in Sodium‐Ion Batteries

**DOI:** 10.1002/anie.202013803

**Published:** 2021-01-08

**Authors:** Le Anh Ma, Andrew J. Naylor, Leif Nyholm, Reza Younesi

**Affiliations:** ^1^ Department of Chemistry—Ångström Laboratory Uppsala University 75121 Uppsala Sweden

**Keywords:** conductive membranes, electrochemistry, electrolytes, interphase chemistry, solid electrolyte interphase

## Abstract

The interfacial reactions in sodium‐ion batteries (SIBs) are not well understood yet. The formation of a stable solid electrolyte interphase (SEI) in SIBs is still challenging due to the higher solubility of the SEI components compared to lithium analogues. This study therefore aims to shed light on the dissolution of SEI influenced by the electrolyte chemistry. By conducting electrochemical tests with extended open circuit pauses, and using surface spectroscopy, we determine the extent of self‐discharge due to SEI dissolution. Instead of using a conventional separator, β‐alumina was used as sodium‐conductive membrane to avoid crosstalk between the working and sodium‐metal counter electrode. The relative capacity loss after a pause of 50 hours in the tested electrolyte systems ranges up to 30 %. The solubility of typical inorganic SEI species like NaF and Na_2_CO_3_ was determined. The electrolytes were then saturated by those SEI species in order to oppose ageing due to the dissolution of the SEI.

## Graphical abstract

The formation and dissolution of the solid electrolyte interphase (SEI) in sodium‐based systems is studied using a new cell setup. As a separator, β‐alumina is employed as a sodium‐conductive membrane to avoid crosstalk between the working electrode and sodium‐metal counter electrode. The SEI dissolution in different time domains is tested and mitigated with new additive strategies. A correlation between SEI dissolution and SEI chemistry is presented.

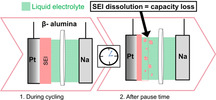

## Introduction

The increasing demand for renewable energy has resulted in an increased interest in sustainable energy conversion technologies, including solar‐ and wind‐based technologies. However, the latter energy sources do not produce energy continuously, which is why stationary energy storage devices connected to the electric grid are required. Lithium‐ion batteries (LIBs) have been widely used in portable applications due to their high energy densities. On the other hand, sodium‐ion batteries (SIBs) based on all abundant elements have attracted attention as SIBs are able to provide more cost‐effective and sustainable stationary energy storage systems.[^1^, ^2^]

To facilitate the implementation of SIBs in practical applications, the long‐term performance of the SIBs still needs to be improved. Hence, high capacity and high voltage electrodes with high stability electrolyte formulations, compatible with both the anode and cathode, need to be developed.[^3^, ^4^, ^5^] The instability of the electrode‐electrolyte interfacial layers is one of the main sources of ageing in SIBs. As most non‐aqueous electrolytes are unstable at the electrochemical potentials of the negative electrodes, the formation of a stable passivation layer known as the solid electrolyte interphase (SEI) is both unavoidable and essential.[^1^, ^6^, ^7^]

In the ideal case, the SEI, which mainly is formed on the first charge/discharge cycle, is electronically insulating, impermeable to the solvent molecules to avoid continuous electrolyte decomposition, and ionically conducting to allow the migration of Na^+^ ions. In addition, the SEI should be insoluble and inert with respect to the electrolyte to avoid parasitic reactions resulting in irreversible capacity losses.[^8^, ^9^, ^10^, ^11^]

Compared to in LIBs, the SEIs formed in Na‐based electrolytes are often reported to be less stable due to the higher solubilities of the SEI components.[^12^, ^13^, ^14^] This can be due to the higher solubilities of most sodium inorganic species present in the SEI when compared to their Li analogues. In aqueous solvents, higher solubilities are thus found for NaF vs. LiF and Na_2_CO_3_ vs. Li_2_CO_3_. In a comparative study, Moshkovich et al. showed that NaClO_4_ yields SEI components which are more soluble in PC than the SEI components obtained from the corresponding Li‐ and K‐salts.^[15]^ Mogensen et al. have likewise demonstrated a higher dissolution rate of the Na‐SEI than the Li‐SEI on carbonaceous anodes in electrolytes containing hexafluorophosphate (PF_6_) dissolved in ethylene carbonate:diethyl carbonate (EC:DEC).^[12]^ To address the ageing mechanisms in SIBs, a more comprehensive understanding of the influence of the electrolyte composition on the SEI formation and dissolution in Na‐based electrolytes is consequently needed.

Several studies have highlighted the influence of the electrolyte components, including the solvent, salt, and additives, on the electrochemical performance and cycle life of SIBs.[^1^, ^16^, ^17^, ^18^] Alcantara et al. showed that a solvent mixture composed of tetrahydrofuran (THF) and EC resulted in better cycling performance compared to 1,2‐dimethoxyethane (DME) or a mixture of EC and dimethyl carbonate (DMC).^[16]^ In another study, in which different Na‐salts and solvent mixtures were tested in a three‐electrode Swagelok cell, it was shown that NaClO_4_ and NaPF_6_ in a mixture of EC and propylene carbonate (PC) gave rise to the best cycling performance.^[1]^ Shkrob et al. demonstrated that the solvents EC and PC continuously form secondary and tertiary radicals which facilitate polymer formation.^[17]^ Using X‐ray photoelectron spectroscopy (XPS), Eshetu et al. showed that NaPF_6_‐based electrolyte systems are more prone to form an organic‐rich SEI layer compared to other salts such as sodium (fluorosulfonyl) (trifluoromethanesulfonyl) imide (NaFTFSI) and sodium perchlorate (NaClO_4_).^[18]^

The additives fluoroethylene carbonate (FEC), difluoroethylene carbonate (DFEC) and vinylene carbonate (VC) have been shown to improve the cycling performance of SIBs. The key role of these additives is to decompose before the other electrolyte components to ensure the formation of a stable passivating SEI on the anode surface.[^19^, ^20^]

Na‐metal is currently used as reference and counter electrode to study Na‐ion systems. However, there are concerns about its stability and reliability during cycling leading to irreproducible results. It has been shown that Na‐metal electrodes form soluble electroactive decomposition products effecting the electrolyte matrix resulting in inaccurate interpretation of electrochemical experiments and identification of SEI composition.[^21^, ^22^, ^23^, ^24^, ^25^, ^26^]

Herein, we describe the results of a systematic study aimed at determining the influence of the SEI dissolution rate on the capacity loss in SIBs, as well as the influence of the electrolyte composition on the solubility of the SEI components. The accessible electrochemical window and the SEI formation and stability in different Na‐based electrolyte systems are evaluated using electrochemical techniques such as potentiometry and chronoamperometry. Furthermore, the SEI compositions are analyzed using synchrotron‐based soft X‐ray photoelectron spectroscopy (SOXPES). To decrease the SEI dissolution rate, the electrolyte is saturated with known SEI species, such as NaF and Na_2_CO_3_.

## Results and Discussion

In conventional alkali‐ion batteries the capacity losses can be explained based on a variety of ageing mechanisms and the capacity loss mechanisms are typically also different for full‐ and half‐cells.[^12^, ^27^, ^28^] As this study only focuses on half‐cells containing Na‐metal electrodes, there are three major sources of the capacity loss: SEI dissolution, ion trapping and volume expansion leading to a cracking of the active material.[^27^, ^28^, ^29^] Since a Na‐metal counter electrode was used, the capacity of the cell was only dependent on the capacity of the Pt electrode, which mainly should stem from the reduction of the electrolyte. The Pt electrodes used in this study remain inert and do not form Na‐alloys or do not face underpotential plating above 0 V vs. Na^+^/Na as no discharge plateau or no high reduction capacity was observed (Supporting information, Figures S1).^[28]^ The measured capacity should hence correspond to the capacity associated with the SEI formation and the replenishment of the SEI in the event of SEI dissolution. Also, the tests (discussed below) clearly show that the observed reduction capacity for re‐formation of SEI is directly related to the volume of the electrolyte.

In this study, two cell‐separator types were employed; that is, cells equipped with a Solupor® separator (C‐SP) and cells containing a β‐alumina (C‐BA) separator (Figure S2). For Na half cells containing conventional separators such as Solupor® (C‐SP), the SEI dissolution could be affected by the crosstalk from the metallic Na electrode.^[30]^ To minimize this effect, solid Na‐conductive β‐alumina discs were used as separators. The influence of the separator (i.e. Solupor® or β‐alumina) on the capacity losses seen for electrolytes composed of 1 M NaPF_6_ dissolved in PC or EC:DEC is shown in Figure S3. When using Solupor® separators, species formed at the Na‐metal electrode can diffuse to the working electrode, influencing chemistry and solubility of the SEI on the metal substrate (Pt). In our case, crosstalk from the Na‐metal electrode had a positive effect on the SEI stability (Figure S3). One possibility could be explained by diffusion of SEI components formed at the Na electrode to the working electrode resulting in an increased concentration of the SEI components in the electrolyte as this should decrease the SEI dissolution rate (see below). However, the existence of crosstalk introduces uncontrollable factors which complicate the fundamental analysis and comparison of the different electrolyte systems. All experiments were therefore carried out with the cell setup C‐BA unless stated otherwise. All the C‐BA cells were disassembled after the cycling and no Na‐deposition was found on the Pt working electrodes.

**Figure 1:**
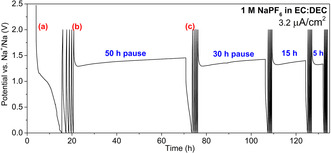
Galvanostatic cycling, between 0.2 mV and 2.0 V vs. Na^+^/Na, combined with pauses after the end of every 5th full cycle. A two‐electrode cell containing a Pt working electrode, β‐alumina separator and Na‐metal counter electrode was used (cell setup C‐BA@Pt). The procedure consisted of five galvanostatic cycles using a constant current of 10 μA, followed by different pause times chosen to illustrate the time‐dependent self‐discharge.

To assess the SEI dissolution rates, galvanostatic cycling and cyclic voltammetry (CV) followed by extended relaxation/pause times were used, as shown in Figure 1 and Figure S1. This type of determination of the remaining capacity after an open circuit period is a useful approach to study the influence of effects yielding capacity losses. Here, we use noble metal working electrodes, that is, Pt electrodes (10 mm diameter) and potentials slightly above 0 V vs. Na^+^/Na to prevent Na‐plating.[^31^, ^32^]

In the experiments, a variety of Na‐based electrolyte systems were used. At low electrode potentials, the electrolyte components undergo reduction at the working electrode, which results in the formation of a passivation layer, the SEI. The first cycle reduction capacity was then taken as a measure of the irreversible capacity due to the SEI formation (Figure 1, point (a) ranging from 2.0 V to 0.2 mV). This wide voltage window was chosen to mimic the experimental conditions used in some studies of anode materials, in which the anode can experience such high voltages at the end of the discharge. Even though the cut‐off potential was low, the charge capacity was low, demonstrating that there was no significant underpotential Na‐deposition. The cell was then cycled for four additional cycles during which the reduction capacities were found to be much smaller indicating that a functioning SEI layer had been formed on the first cycle. After five full cycles, a pause of 50, 30, 15 or 5 hours without current flow is applied (Figure 1, point (b)), before the cycling is continued with the next reduction step (Figure 1, point (c)). The reduction capacity was then found to be larger, for example up to 30 times larger after 50 h pause, than the reduction capacity of the fifth cycle prior to the pause. Although longer pause times could have been used, the abovementioned pauses were found to be sufficient to illustrate the capacity loss phenomenon. Shorter pauses than five hours did, however, not yield any significant capacity losses. The reverse order of pause times was also tested. Figure S4 shows that the sum of all the capacity losses after all the pauses are similar for both cases whether we start from short or long pause times. The results also indicate that the applied pause times are not enough to saturate the electrolyte with SEI species as there is still some capacity loss even after applying several long pause times. This is expected given that the amount of SEI formed on the low‐surface‐area Pt substrate is quite low, so that even if all SEI species are dissolved into the electrolyte, the volume of the electrolyte (150 μL) is still too large to be saturated. As the fifth cycle was stopped at 2.0 V vs. Na^+^/Na, the potential of the working electrode should be higher than of the electrolyte. When pausing the cell under open circuit voltage (OCV), the electrode potential decays to the electrolyte potential, inducing the sudden potential drop to 1.3 V vs. Na^+^/Na observed in Figure 1, point (b). In the absence of any redox capacity, the potential drop can be associated with discharge of an electric double layer. The slight increase seen in the cell potential during the pause time suggests that the SEI is not sufficiently stable and dissolves in the electrolyte.

If the capacity loss was caused by SEI dissolution, the electrolyte volume should affect the magnitude of the capacity loss significantly as a larger electrolyte volume should give rise to lower concentrations of the SEI components in the electrolyte. A larger electrolyte volume should thus lead to a larger capacity loss due to SEI dissolution, according to Le Chatelier's principle.

**Figure 2:**
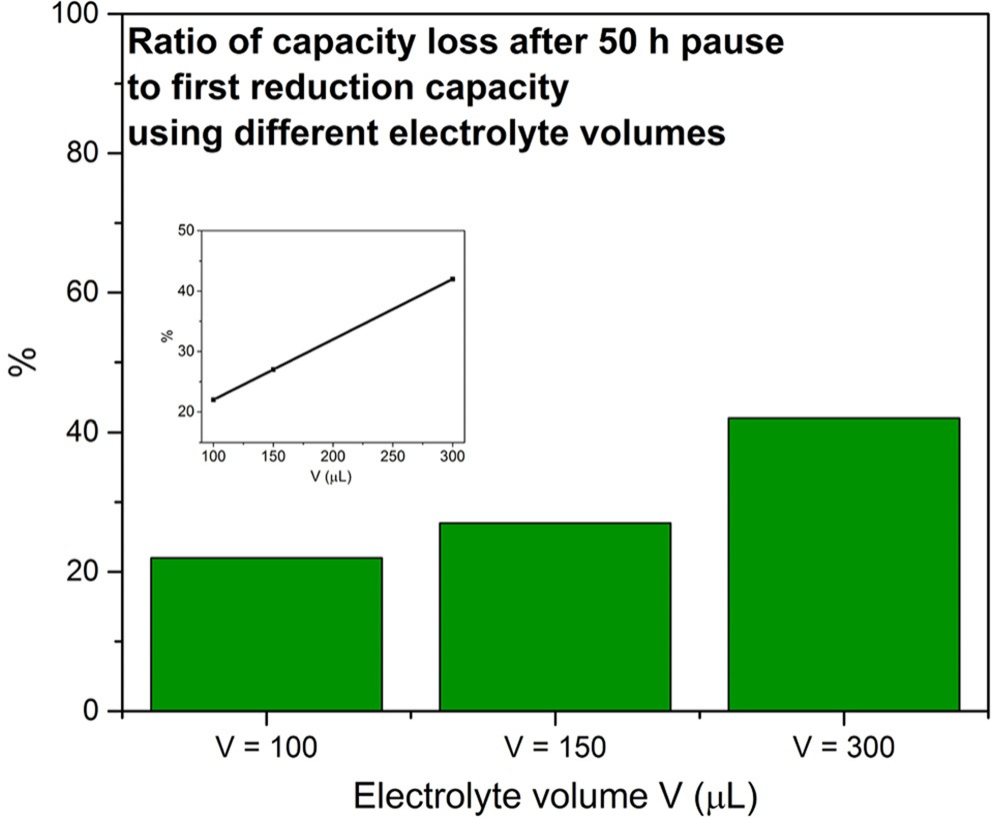
The capacity loss ratios after a 50 h pause (with respect to the first reduction capacity for the SEI formation) for different electrolyte volumes. When using an electrolyte volume of 300 μL the capacity loss after 50 h pause was thus 42 % of the initial capacity consumption for the SEI formation on the first cycle. The observed capacity loss was clearly strongly affected by the electrolyte volume. The inset shows the linear correlation between the relative capacity loss after a 50 h pause and the electrolyte volume.

The influence of the electrolyte volume on the SEI dissolution rate was studied. Figure 2 and Figure S5 clearly show that the magnitude of the capacity loss depended on the employed electrolyte volume. This is in good agreement with the SEI‐dissolution hypothesis and confirms that the measured capacity is not related to any other phenomena like Na trapping, alloying or underpotential plating. The dependence of the capacity loss on the electrolyte volume also shows that deposition of elemental Na on the electrode is unlikely to be the cause of the observed capacity loss. The results therefore indicate that the capacity loss was mainly due to SEI dissolution and that the SEI capacity loss could correspond to up to 42 % of the first cycle capacity when using an electrolyte volume of 300 μL (Figure 2). As the capacity loss was practically linearly dependent on the pause time, it is also reasonable to assume that the SEI dissolution rate was affected by natural convection.

### Solvent chemistry and non‐conventional additives

**Figure 3:**
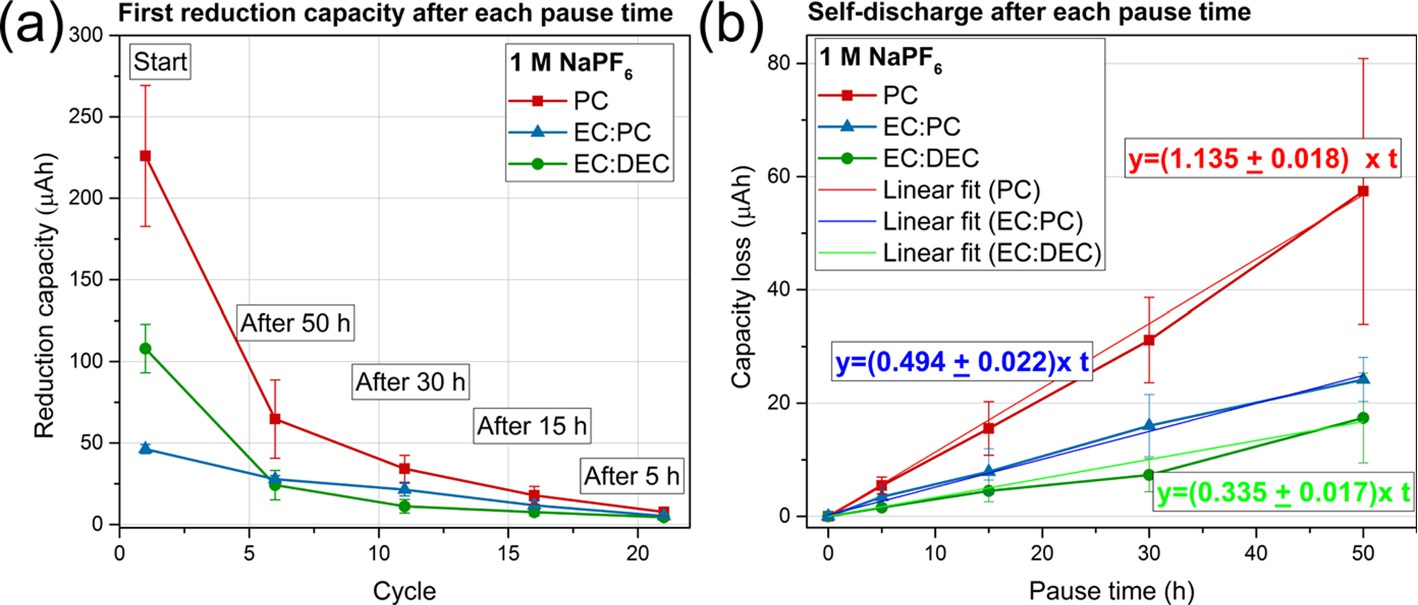
High‐precision galvanostatic cycling results obtained with electrolytes containing 1 M NaPF_6_ in PC, EC:PC or EC:DEC using the C‐BA@Pt cell setup. a) The reduction capacity obtained after each pause time as a function of the cycle number. b) The capacity loss as a function of the pause time.

The influence of the solvent on the reduction capacity (i.e. the SEI formation charge) is presented in Figure 3 a, whereas in Figure 3 b the differences between the reduction capacity before and after each pause time are presented. Figure 3 a,b is based on data generated using galvanostatic measurements at 30 °C, as shown in Figure 1. The electrolytes were 1 M NaPF_6_ in PC, EC:DEC or EC:PC, while the error bars in the plots correspond to the standard deviation from the average value of three replicate cells. Figure 3 a displays the first reduction capacity associated with the SEI formation and the following reduction capacities after a pause as a function of the number of cycles, following the cycling procedure described in Figure 1. Generally, the capacity associated with the SEI formation process and the additional reduction capacity required after the open circuit pause periods were relatively small, that is, in the range of μAh, which is why the experiments were carried out employing high‐precision galvanostatic cycling. Lindgren et al.^[27]^ estimated the capacity needed to form a 20 nm thick SEI to be around 0.052 mAh on nano‐Si composite electrodes, which is consistent with our values ranging from 0.05–0.25 mAh on Pt.

While a similar trend was seen for EC:PC and EC:DEC, the PC results indicated a higher initial SEI formation capacity. Generally, the choice of electrolyte solvent affected the SEI related capacity during the first 20 cycles. After that, the difference in capacity consumption was negligible, suggesting that the electrolyte gradually became saturated with soluble SEI components. The initial reduction capacity of EC:PC (50 μAh), followed by EC:DEC (110 μAh) were significantly lower than that for PC (230 μAh). The accumulated reduction capacity plots showed that PC exhibited a higher reduction capacity than EC:PC and EC:DEC (Figure S6a). Based on the curves, it is reasonable to assume that the low reduction capacities were obtained with the EC:PC and EC:DEC based electrolytes. In Figure 3 b, the capacity loss is shown as a function of the pause time for the same electrolytes. In agreement with the results in Figure 2, a linear increase in the capacity loss is seen when increasing the duration of the pause, indicating that the effect was due to SEI dissolution. The SEI dissolution rates were found to be approximately 1.1, 0.5 and 0.3 μAh h^−1^ in PC, EC:PC and EC:DEC, respectively. The capacity losses due to the SEI dissolution during the pause of 50 hours corresponded to about 20 to 50 % of the initial reduction capacities for the three electrolytes (Figure S6b). The present results consequently indicate that, of the three tested electrolytes, EC:DEC exhibited the smallest initial SEI formation capacity as well as the lowest SEI dissolution rate. This information could be used to predict capacity losses due to SEI formation and dissolution in Na‐ion batteries containing these types of electrolytes.

### Mitigating SEI dissolution

One approach to mitigate the SEI dissolution could be to saturate the electrolyte with typical SEI components. This shifts the concentration equilibrium to make those SEI species less soluble in the electrolyte. The solubility of some salts should propose an approach on how to determine the SEI solubility. However, we are hinting towards it, by mentioning the ICP‐OES results and its correlation with the SEI dissolution (*solubilities of NaF and Na_2_CO_3_ in different solvents)*.

For electrolytes composed of NaPF_6_ and carbonate‐based solvents, NaF and Na_2_CO_3_ are known to be present in the SEI layer.^[33]^ Experiments were therefore carried out in which the electrolytes were saturated with NaF or Na_2_CO_3_ in an attempt to decrease the SEI dissolution rates.

**Figure 4:**
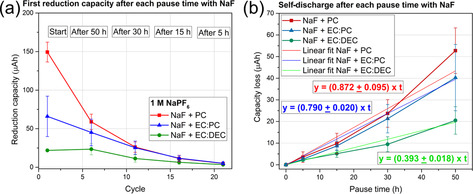
High‐precision galvanostatic cycling results obtained with electrolytes comprising 1 M NaPF_6_ in PC, EC:PC or EC:DEC, saturated with NaF. a) The reduction capacity obtained after each pause time as a function of the cycle number. b) The capacity loss as a function of the pause time.

As shown in Figure 4 a, the addition of NaF appears to decrease the first reduction capacity for the PC and EC:DEC systems, indicating that NaF is an effective additive for suppressing SEI formation in the studied electrolytes. However, for EC:PC no significant change could be observed, taking the error into consideration. However, for PC and EC:DEC systems, this indicates that a high concentration of NaF in the electrolyte resulted in a decreased NaF SEI dissolution rate, which decreased the overall SEI dissolution rate. In the EC:DEC electrolyte containing NaF, the initial SEI capacity is only about 25 μAh which should be compared to about 110 μAh obtained without the addition of NaF, shown in Figure 3 a. It is reasonable to assume that the EC:PC and EC:DEC electrolytes had low SEI consumption, whereas PC showed a relatively high reduction capacity. This conclusion is further supported by the accumulated reduction capacities (Figure S7a), which showed that EC:DEC exhibited the lowest reduction capacities after NaF addition, throughout the cycling. Also, the dissolution rate for EC:DEC is significantly smaller than in EC:PC and PC. In general, the results suggest that the addition of NaF resulted in a decreased initial SEI formation capacity for PC and EC:DEC by 33 % and by 77 %, respectively, whereas an increase by 60 % was seen in EC:PC.

In accordance with the results in Figure 3 b, the lowest SEI dissolution rate (i.e. 0.4 μAh h^−1^) is observed with the EC:DEC electrolyte even after adding NaF (Figure 4 b). This rate was, however, not significantly different from the rate of 0.3 μAh h^−1^ obtained in the absence of NaF. In PC and EC:PC, the SEI dissolution rates were quite similar, that is, 0.9 μAh h^−1^ in PC and 0.8 μAh h^−1^ in EC:PC (Figure 4 b). The addition of NaF thus decreased the SEI dissolution rate in PC by 19 %, while an increase of 38 % was seen for EC:PC. Overall, the most promising NaF addition results were hence obtained when using EC:DEC.

**Figure 5:**
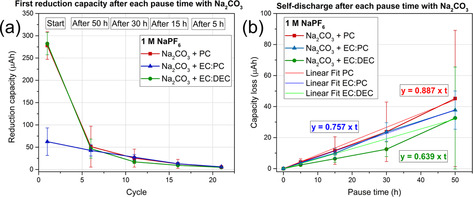
High‐precision galvanostatic cycling results obtained with electrolytes comprising 1 M NaPF_6_ in PC, EC:PC and EC:DEC and saturated with Na_2_CO_3_. a) The reduction capacity obtained after each pause time as a function of the cycle number. b) The capacity loss as a function of the pause time.

The saturation of PC and EC:DEC based electrolytes with Na_2_CO_3_, however, showed a detrimental effect as the first reduction capacity of ca. 280 μAh (Figure 5 a) was higher than the values of 230 μAh and 110 μAh obtained for PC and EC:DEC not containing any added Na_2_CO_3_ (Figure 3). For EC:PC, the addition of Na_2_CO_3_, on the contrary, led to a decrease in the initial SEI capacity (i.e. 60 compared to 130 μAh). These effects are also consistent with the accumulated reduction capacities shown in Figure S6b. As seen in Figure 5 b, there are no significant differences between the SEI dissolution rates: 0.9, 0.8 and 0.6 μAh h^−1^ for PC, EC:PC and EC:DEC, respectively. This means that the SEI dissolution rate in the PC and EC:PC cases were little affected by the addition of Na_2_CO_3_, whereas the rate in the EC:DEC case increased by about 91 %. Saturation of the electrolyte with Na_2_CO_3_ does therefore not constitute a promising approach to decrease the SEI‐related capacity losses in the investigated electrolytes.

For the electrolyte systems tested so far, it is generally clear that a lower capacity loss rate was seen with EC:DEC as the solvent than with PC or EC:PC. Even though there clearly are many other inorganic and organic components in the SEI influencing its formation and stability, the present results constitute the first approach to study the influence of individual inorganic SEI species on the SEI formation capacity and dissolution rates. Similar tests should naturally also include organic SEI species, which are unfortunately more difficult to characterize and to prepare ex‐situ. One possibility would, however, be to pre‐treat the electrolyte with for example, Na.

**Figure 6:**
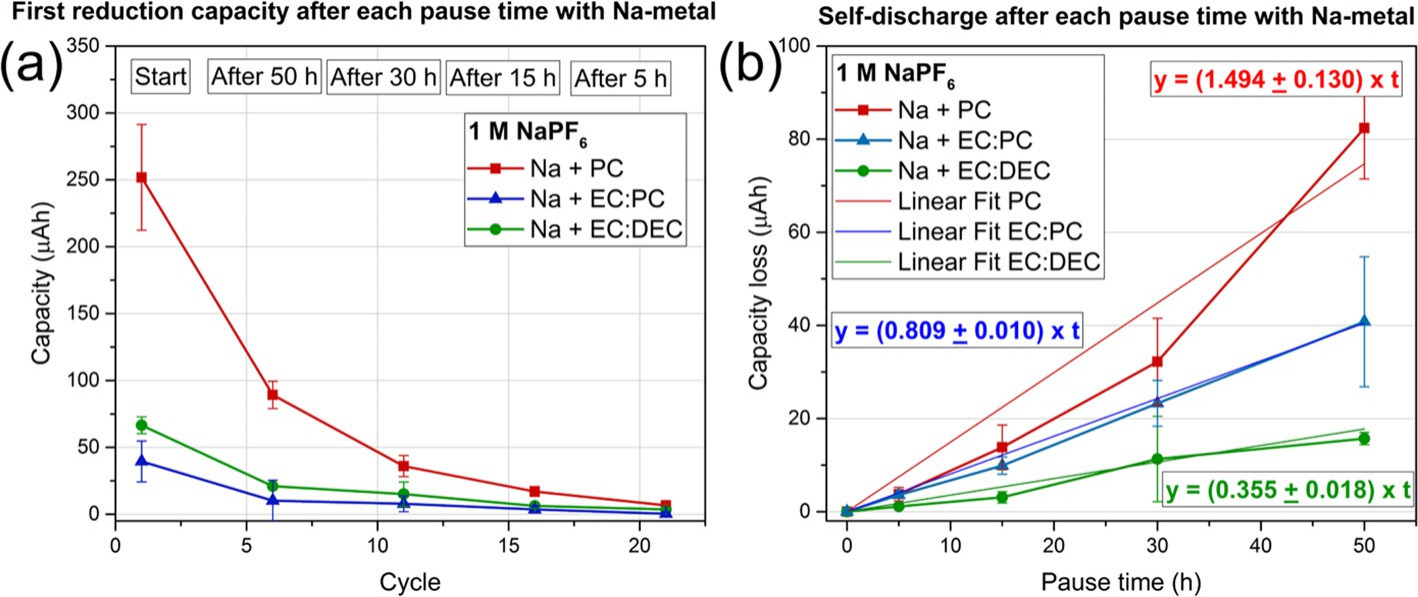
High‐precision galvanostatic cycling results obtained with electrolytes comprising 1 M NaPF_6_ in PC, EC:PC or EC:DEC, exposed to Na metal for 24 h. a) The reduction capacity obtained after each pause time as a function of the cycle number. b) The capacity loss as a function of the pause time.

As mentioned before, the presence of a Na‐metal anode in the cell results in less SEI dissolution. This could be due to the formed organic species at the Na‐metal, which continuously dissolve and saturate the electrolyte. Therefore, the organic SEI species formed on the Pt electrode do not undergo dissolution. To generate organic SEI species directly in the electrolyte, the electrolytes were hence first exposed to a cubic piece of metallic Na (ca. 30 mg per 500 μL electrolyte volume). Due to the Na reacting with the electrolyte, an increase in the concentration of the organic SEI species in the electrolyte was expected. As can be seen by comparing Figure 3 a and Figure 6 a, the trend in the first reduction capacity is, however, the same as that obtained without using any Na. The largest first reduction capacity (250 μAh) was again seen for PC, while there was no significant difference between the values obtained for EC:DEC and EC:PC (70 and 30 μAh). A corresponding comparison of Figure 3 b and Figure 6 b also indicates that the exposure of the electrolytes to Na‐metal did not change the trend of capacity loss rate between the electrolytes compared to the control electrolytes.

This indicates that the generated concentrations of the electrolyte reduction products were too low to affect the SEI formation and SEI dissolution processes. This could be due to the relatively small surface area of the cube, which most likely was rapidly passivated when immersed in the electrolyte. Additionally, the presence of Na‐metal in the electrolyte should also mimic the presence of a Na‐metal electrode in a half cell and one would expect a decrease in SEI dissolution as observed in Figure S3. However, this is not the case, which could be, as mentioned before, due to the small surface area of Na‐metal which is passivated instantly after exposing to electrolyte. Furthermore, in a half‐cell setup with Solupor® separator, Na‐metal would be continuously electrochemically active during cycling and saturate the electrolyte with organic species, which is not the case in our tested systems (C‐BA@Pt).

It can thus be concluded that the trends regarding the reduction capacities and capacity loss rates remain the same after exposing the electrolyte to Na‐metal.

**Figure 7:**
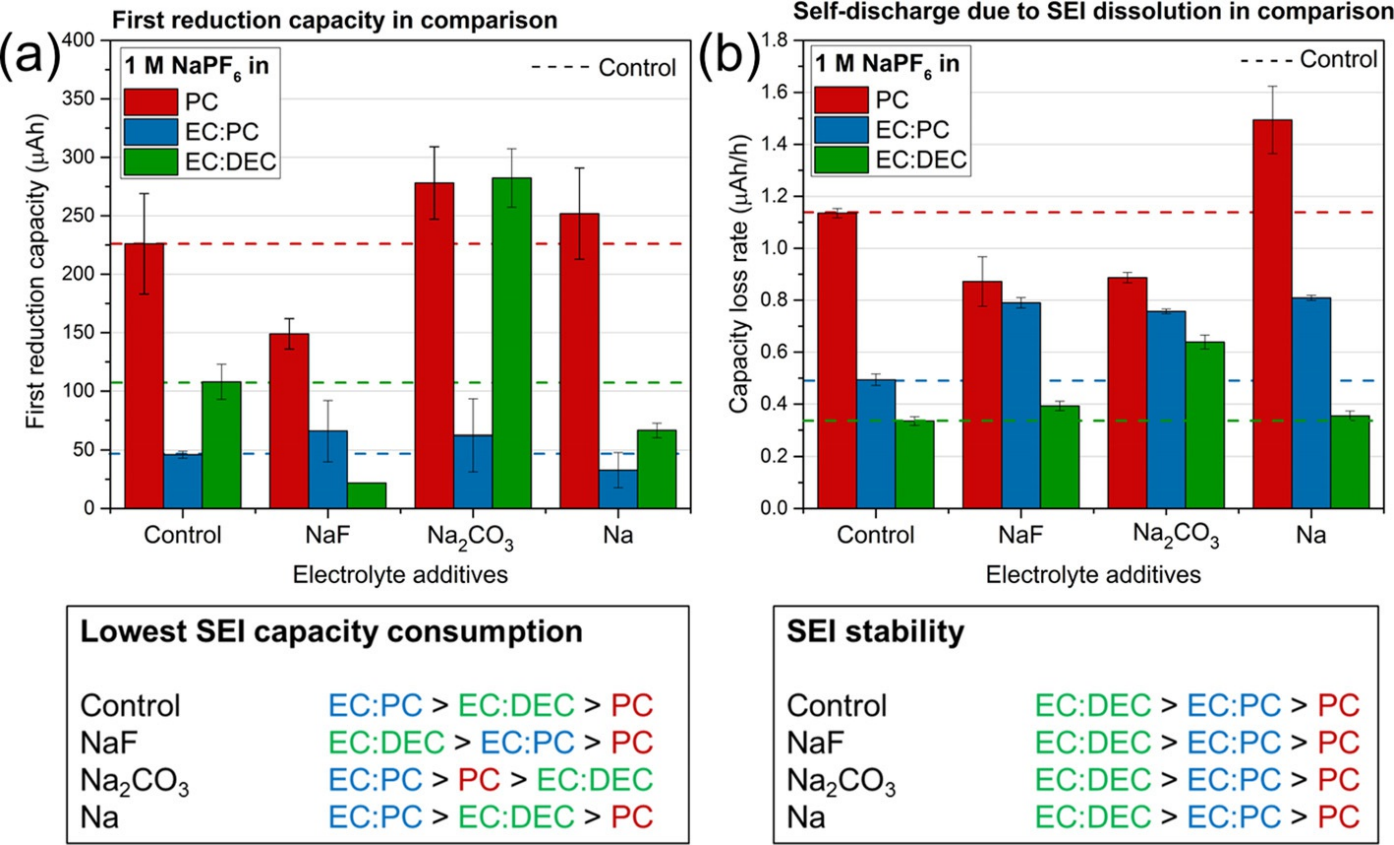
Summary of all the results obtained prior to and after adding NaF, Na_2_CO_3_ or Na to the three electrolytes, that is, 1 M NaPF_6_ in PC, EC:DEC and EC:PC, respectively. a) First reduction capacities and a corresponding ranking of the SEI capacity consumptions. b) Capacity loss rates and a corresponding ranking of the capacity loss rates (i.e. the SEI dissolution rates).

Summaries of the results for the three electrolytes, in the presence and absence of the three additives, are given in Figure 7 and Figure S8 as well as Tables S1 and S2 in the Supporting Information. The data indicate that the lowest first reduction capacity was obtained in the EC:DEC based electrolytes saturated with NaF. Also, the addition of NaF showed clear impact on decreasing the first cycle reduction capacity and also the rate of capacity loss for PC electrolytes. Addition of Na_2_CO_3_ had detrimental effect on the first reduction capacity in all three electrolyte systems, and the rate of capacity loss of EC:DEC and EC:PC electrolytes. However, Na_2_CO_3_ showed beneficial influence on the rate of capacity loss for PC electrolyte. The exposure of the electrolyte to Na‐metal (a cube of Na‐metal for short exposure) show no beneficial effect on the rate of capacity loss of any of the electrolyte.

Overall, the lowest SEI dissolution rates were found when using the NaPF_6_‐EC:DEC electrolytes. In general EC:DEC electrolyte systems showed a low capacity loss rate. This indicates that a more stable SEI was formed in the EC:DEC based electrolytes than in EC:PC and PC.

The lower first reduction capacities (i.e. SEI formation capacity) for 1 M NaPF_6_ dissolved in EC:DEC (100 μAh) and EC:PC (50 μAh) thus suggest the formation of a relatively stable SEI layer with a lower capacity loss rate of 0.3–0.5 μAh h^−1^, whereas the SEI layers formed in the corresponding PC based electrolytes (220 μAh) appear to dissolve faster (1.1 μAh h^−1^). This leads to higher first reduction capacities as well as SEI dissolution rates during the imposed open circuit pauses. A low first reduction capacity is clearly desirable to minimize the initial capacity loss, while a stable (i.e. poorly soluble) SEI is required to decrease the capacity loss during cycling and storage. The results of this study consequently show that the first reduction capacity and the SEI stability are interconnected for the electrolytes studied here.

### Solubilities of NaF and Na_2_CO_3_ in different solvents

It has often been stated that the SEI layer consists of both inorganic and organic species containing the employed alkali ion. Generally, the SEI stability depends on the solubility of the SEI components formed in each solvent system.^[34]^ While comparisons with the solubilities in water can provide valuable information, such an approach may unfortunately not be valid for all alkali salts in all solvents. One needs therefore to determine the solubilities in the relevant solvents. Inductively coupled plasma optical emission spectroscopy (ICP‐OES) measurements were therefore carried out to measure the Li^+^ or Na^+^ concentrations in saturated solutions of LiF, Li_2_CO_3_, NaF and Na_2_CO_3_ in PC and EC:DEC. The results in Table 1 show different trends for the Li‐ and Na‐salts in the same solvent system. Note that the differences between the solubilities for NaF and Na_2_CO_3_ are quite unlike those in water. According to the ICP‐OES results, the Li‐salts have significantly lower solubilities in both solvent systems compared to the Na‐salts. This is important as it can explain the higher stabilities seen for Li‐based SEI layers compared to the corresponding Na‐based ones. It is also seen that while NaF is considerably less soluble than Na_2_CO_3_ in water, NaF is only slightly less soluble than Na_2_CO_3_ in EC:DEC and it is slightly more soluble than Na_2_CO_3_ in PC. It is, however, clear that both NaF and Na_2_CO_3_ are less soluble in EC:DEC than in PC.

**Table 1:** LiF, Li_2_CO_3_, NaF and Na_2_CO_3_ solubilities in H_2_O (literature values from Ref. [35]), PC and EC:DEC (measured with ICP‐OES).

	Solubility		
	In H_2_O [g L^−1^]^[35]^	In PC [mg L^−1^]	In EC:DEC [mg L^−1^]
LiF	1.34	0.157	0.066
Li_2_CO_3_	13.0	0.160	0.135
NaF	41.3	8.617	3.057
Na_2_CO_3_	307	6.603	3.648

The electrochemical analyses showed that the SEI capacity loss rate in EC:DEC was lower than in PC (Figure 7 b). This implies that smaller amounts of NaF and Na_2_CO_3_ can dissolve from the SEI into the EC:DEC electrolyte, compared to in PC, in agreement with the ICP‐OES results. The addition of NaF and Na_2_CO_3_ as additives to the electrolyte should hence be more effective for a NaPF_6_‐PC electrolyte than for a NaPF_6_‐EC:DEC electrolyte resulting in a decreased self‐discharge rate due to a lower SEI dissolution rate (Figure 7 b).

### SOXPES studies of the SEI layers

To shed light on the SEI chemistry in reference electrolytes based on EC:DEC and PC, synchrotron‐based soft X‐ray photoelectron spectroscopy (SOXPES) with photon energy of 1090 eV was used (Figures S9–S11).^[36]^ The CVs in Figures S1 and S12 show a large reduction current due to solvent and water reduction. The amount of water in the electrolyte can affect the contents of inorganic and organic species in the SEI dependent on what solvent system was used. However, the focus of our work, is to show the SEI dissolution for our studied electrolyte systems.

**Figure 8:**
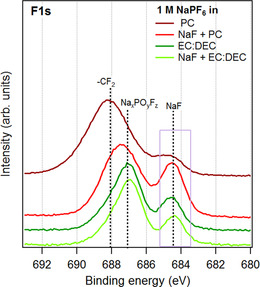
F 1s SOXPES spectra recorded for Cu electrodes after 10 CV cycles between 0.1 and 2.0 V in two different electrolyte systems in the presence and absence of NaF. The CV experiment was stopped at 2.0 V after the 10th cycle. The NaF addition resulted in a higher NaF SEI content in the NaPF_6_‐PC system, whereas no significant change was seen in the NaPF_6_‐EC:DEC case.

To understand the NaF addition effect, the SEI composition after the first reduction with and without the NaF additive in the electrolyte was analyzed by SOXPES (Figure 8; Figure S11). In the EC:DEC case there was no significant difference between the compositions of the SEI layers generated in the presence and absence of NaF. A difference between the compositions of the SEI layers was, however, seen when using PC as the solvent. In this case, the NaF addition resulted in an SEI layer with a higher NaF content, indicating that the added NaF induced the formation or incorporation of more NaF in the SEI. As the electrochemical results demonstrated that the addition of NaF decreased the self‐discharge rate in PC (Figure 7 b), it is reasonable to assume that this effect was linked to the increased NaF content in the SEI layer and hence the more inorganic nature of the SEI layer indicated by the XPS results.

This study constitutes the first step towards understanding the connection between SEI dissolution and the capacity losses seen during the cycling of sodium‐ion batteries in carbonate‐based electrolytes. It is clear that the SEI dissolution involves many more species than the model compounds NaF and Na_2_CO_3_ used here. Additional experiments are required to determine the solubilities for many other SEI components, including the organic SEI species. A variety of electrolyte reduction products hence need to be identified to enable an optimization of the SEI stability.[^8^, ^33^, ^37^, ^38^, ^39^]

## Conclusion

A comparative analysis of the SEI formation and dissolution in three Na‐based electrolytes was performed. The capacity consumed to form SEI, the SEI composition and the capacity loss due to the dissolution of the SEI all depend on the electrolyte chemistry including the solvent, salt, and additives.

SEI formed in NaPF_6_‐PC resulted in a higher dissolution than in NaPF_6_‐EC:PC and NaPF_6_‐EC:DEC. One reason why the SEI formed in PC had a lower stability could be that inorganic SEI components such as NaF and Na_2_CO_3_ are more soluble in PC than in EC:DEC. To oppose this dissolution, NaF and Na_2_CO_3_ were used as electrolyte additives to saturate the electrolytes.

Strategies and methods in this work can add substantially to our current knowledge about mitigating SEI dissolution as an ageing mechanism in Na‐ion batteries. In our case, one should aim for an SEI layer based on species with low solubilities in the employed solvent. For example, in solvent systems such as EC:DEC and PC, a more inorganic SEI should be obtained for a longer SEI stability during pause time. Adding NaF to NaPF_6_ in PC increased the NaF content in the SEI, resulting in a more inorganic and stable SEI. The other additive was Na_2_CO_3_, which was added to PC, resulting in a lower self‐discharge rate than without the carbonate additive (Figure 7 b). This type of additive opens up possibilities for broader electrolyte additive research. A future step could be to design electrolytes that promote SEI layers formed from low‐solubility species that also passivate the electrode effectively.

## Conflict of interest

The authors declare no conflict of interest.

## Supporting information

As a service to our authors and readers, this journal provides supporting information supplied by the authors. Such materials are peer reviewed and may be re‐organized for online delivery, but are not copy‐edited or typeset. Technical support issues arising from supporting information (other than missing files) should be addressed to the authors.

Supplementary
