# Supplementary material for: Strategies for Mitigating Dissolution of Solid Electrolyte Interphases in Sodium‐Ion Batteries
Source: Angew Chem Int Ed Engl. 2021 Jan 8;60(9):4855–63. doi: 10.1002/anie.202013803 (PMC7986800; doi:10.1002/anie.202013803)
Supplement: Supplementary file 1 — Supplementary [file ANIE-60-4855-s001.pdf]

Angewandte Chemie
Eine Zeitschrift der Gesellschaft Deutscher Chemiker

Supporting Information

Strategies for Mitigating Dissolution of Solid Electrolyte Interphases in Sodium-Ion Batteries

*Le Anh Ma, Andrew J. Naylor, Leif Nyholm, and Reza Younesi**

anie_202013803_sm_miscellaneous_information.pdf

Supporting Information

Table of Contents

Experimental Procedures Page 2
Results and Discussion Page 3
Authors Contribution Page 11
References Page 11

Experimental Procedures

Cell setup

Different cell setups were used to study the SEI formation in three Na-based carbonate electrolyte systems (Figure S3). Platinum (Pt) discs (10 mm diameter, 0.1 mm thickness, Goodfellow) and copper foil (13 mm diameter) were cycled against Na-metal (13 mm diameter) in polyethylene coated aluminium pouch cells using the following electrolytes: 1 M $NaPF_6$ (Stella) dissolved in PC (BASF), EC (BASF) and DEC (BASF), respectively. The EC:DEC and EC:PC electrolytes were 1:1 volume-based mixtures. The additives NaF and Na_2CO_3 (both Sigma Aldrich) were added to the electrolyte until precipitation occurred (i.e. until a concentration of 10 mg/ml is reached). For Na metal addition, a sodium cube was dried thoroughly with paper towels. Inside the Ar-filled box, the oxide layer on the cube was cut off with a scalpel. The metallic cube was then cut into smaller pieces (30 mg). The 30 mg Na-metal cube was added to 500 μL electrolyte (60 mg/ml) after which the reaction was allowed to proceed during 24 h. The cells were assembled in an Ar-filled glovebox (<1 ppm H_2O and <1 ppm O_2). As a separator, either a Solupor® (Figure S3: C-SP, Lydall – 3PO7A) or a β-alumina (Figure S3: C-BA, Ionotech, 20 mm diameter, 1 mm thickness) separator was used. The cell setup notation C-SP@Cu correspond to cells with Solupor® separators used together with Cu electrodes, whereas the cell setup notation C-BA@Pt corresponds to cells with β-alumina separators and Pt electrodes. The specific cell setup used is indicated in the figures. The electrolyte volume was 150 μL unless stated otherwise.

Electrochemical measurements

All CV measurements were performed on a Bio-Logic VMP2 instrument using a scan rate of 0.5 mV/s. All galvanostatic testing was conducted with a Novonix high-precision cycler system using constant current of 10 μA.

a) In the high-precision galvanostatic cycling (see Figures 1, 3 ,4, 5, 6 and 7), in which the cell setup C-BA@Pt was used, the cell was cycled from 0.2 mV to 2.0 V vs. Na^+/Na for 5 cycles, before pausing for 50 h. The same step was repeated with pause times of 30 h, 15 h and 5 h. For each measurement series, three identical cells were employed. The error bars represent the standard deviation from the average value from the three identical cells. The lower cut-off potential of 0.2 mV, which is slightly above 0 V vs. Na^+/Na, was used. The voltage range was chosen based on the used potentials to test anode materials (ca. 0 – 2.0 V vs. Na^+/Na). The experimental setup was inspired by that described by Mogensen et al.[1] Their SEI stability studies involving hard carbon anode materials were performed in $NaPF_6$-EC:DEC using different pause times.

b) In the high-precision galvanostatic cycling in Figures 2 and S2, the cell setup C-BA@Pt was used to determine the SEI dissolution rates in different electrolyte volumes (100 μL, 150 μL, 300 μL). The cells were cycled from 0.1-2.0 V vs. Na^+/Na for 5 cycles, stopped at 0.1 V, paused for 50 h and then cycled again (first discharge and then charge). The same procedure was repeated with pause times of 30 h, 15 h and 5h.

c) In the cyclic voltammetry (CV) measurements in Figure S1, the cell setup C-BA@Pt was used and cells were cycled from 0.05 V to 2.0 V vs. Na^+/Na for 10 cycles and then paused for 15 hours and then cycled again for 10 cycles. The reason why a lower cut-off potential was used in this experiment was the higher resistance of the β-alumina separator compared to that for the Solupor® separator.

d) The XPS results in Figure 9, S8, S9, S10 and S11, were obtained with the cell setup C-SP@Cu, including a copper foil (13 mm diameter) electrode and a Solupor® separator. The cells were cycled using CV between 0.1 and 2.0 V vs. Na^+/Na for 10 cycles, stopped at 2.0 V vs. Na^+/Na and then disassembled prior to the XPS measurements. The intensities were corrected using the core level photoionization cross-sections and the relative XPS intensities were compared for each sample. Although copper foils have a native copper oxide layer which can undergo conversion reactions in Na half cells generating Cu and Na_2O, a Na_2O peak could not be detected with XPS (see Figure S9). The influence of the CuO and Cu_2O conversion reactions can be disregarded since this experiment was only carried out to study the composition of the generated SEI layer. The electrochemical results for the C-SP@Cu cells (see Figure S11) were not included in the electrochemical analysis as their purpose was merely to enable an XPS study of the composition of the SEI layer.

e) In the comparison of the Solupor® and β-alumina separators, the same procedure was used as described in a) or b). For comparability, the cell setups C-SP@Pt and C-BA@Pt were used. The pause times, which were arbitrarily

selected, only served to show the qualitative differences between the C-SP and C-BA setups. As seen in Figure S11, there were no significant redox peaks due to the native copper oxide.

General note: All salts and additives were dried under vacuum at 120°C for 24h, and hence had a low water content. Moreover, at low electrolyte potential, the residual water will be depleted due to reduction and reactions with the electrolyte salt or Na metal. The water reduction can be described by following reaction:

$$\mathrm{H_2O} + 2\mathrm{e}^- \rightarrow H_2 + O^{2-}$$

Thus, the water reduction should give rise to H_2 and O^{2-}, which can further react to other species. Therefore, the water content can affect the results and lead to different SEI components. Possible salt impurities should affect the whole test series and should hence not influence the comparisons of the electrolytes containing the same concentration of a salt. The voltammograms shown in Figure S1 suggest that the water content in the electrolytes can be different. However, this work focuses on SEI dissolution observed in our tested electrolyte systems and how it can be mitigated.

Materials characterizations
XPS. Cu discs (10mm diameter) were cycled (as described in *Electrochemical measurements, d*) against Na-metal using Solupor® separator. The cells were stopped at 2.0 V vs. Na^+/Na and disassembled in an Ar-filled glovebox (<2 ppm H_2O and <2 ppm O_2). The Cu foils were then cleaned with 0.5 ml dimethyl carbonate (DMC, Sigma Aldrich) and mounted on carbon tape in a glass vial, vacuum sealed in polyethylene-coated aluminium pouch bags and transported to the synchrotron facility. Photoelectron spectroscopy of the material surface and bulk was performed at the I09 beamline at Diamond Light Source (Oxfordshire, UK). The tested electrodes were fixed with carbon tape to an omicron-type copper plate at the end-station at the synchrotron.

Soft photoelectron spectroscopy (SOXPES) was performed by using an excitation energy of 1090 eV at a branch of the beam line with a plane grating monochromator. The measured sample spot was estimated to be ca. 300 μm long and up to 1 mm wide. During the measurements, no charge neutraliser was used. To record the spectra a hemispherical VG Scienta EW4000 analyser, set to a pass energy of 50 eV was utilised. The analysis of the spectra was conducted using Igor Pro. To calibrate the binding energies in the recorded spectra the peak originating from the C-C bonds in the formed SEI set at 285 eV in the C 1s spectra was used.

ICP-OES. The solubilities of NaF, LiF, Na_2CO_3 and Li_2CO_3 in PC and EC:DEC were determined with ICP-OES using an Avio 200 instrument. Each one of the abovementioned compounds were added to PC and EC:DEC (1:1) until precipitation. The mixtures were then filtered with a 0.5 μm syringe filter evaporated in an oven at 100°C. Water was added to dilute the residual salt and the concentration of the elements were then measured using ICP-OES. A multi-standard solution (Multi-Element Calibration Standard 3, Perkin Elmer, 5% HNO_3) was used to calibrate the instrument. The Li and Na concentrations in the samples were then determined using the Li (670.783 nm) and Na (589.592 nm) lines.

Results and discussion

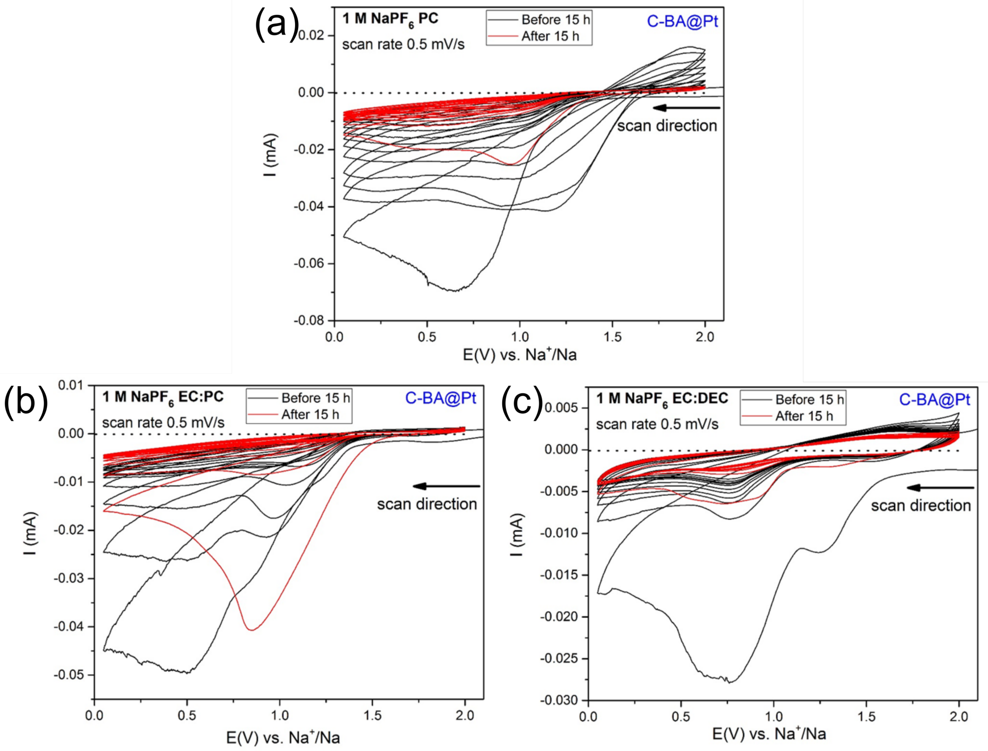

Figure S1. Cyclic voltammograms recorded at a scan rate of 0.5 mV/s with the C-BA cell setup using Pt-electrodes (C-BA@Pt) in (a) 1 M $NaPF_6$ in PC, (b) 1 M $NaPF_6$ in EC:PC and (c) 1 M $NaPF_6$ in EC:DEC. The scan was started at the OCV (ca. 2.0 V vs. Na^+/Na) and the scan direction is shown in the voltammograms. The cycling range was from 0.05 V to 2.0 V vs. Na^+/Na. The cell was cycled for 10 cycles, paused for 15 hours and then cycled again for ten cycles. The low cut-off potential of 0.05 V vs. Na^+/Na was used to prevent Na-plating. The large reduction current on the first cycle can be explained by the reduction of solvent and water present in the solvent. The water reduction should occur at ca. 1.1 V *vs.* Na^+/Na. Hence, based on these results, the water contents can be different in each solvent system which can affect the SEI chemistry.

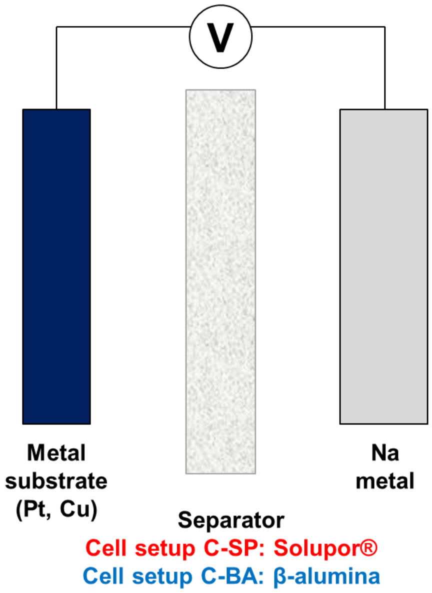

Figure S2. Cell setups used in the investigations of the SEI dissolution effect. The species generated at the Na-metal anode could diffuse to the working electrode and thus influence the electrochemical measurements and the composition of the SEI layer on the working electrode. By using a Na-conductive β-alumina separator, this effect can be avoided.

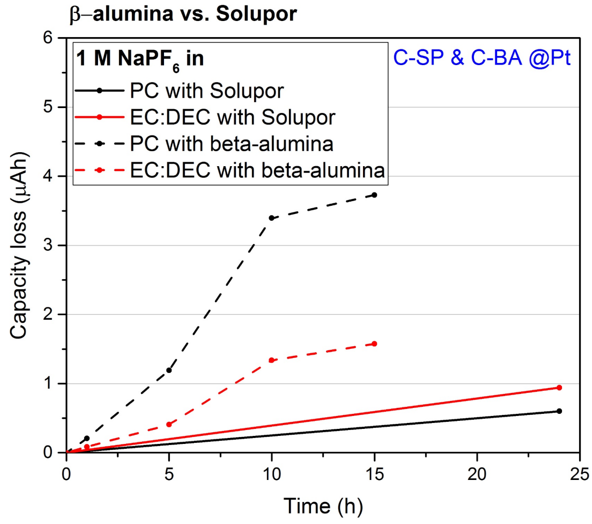

Figure S3. Plot of the capacity loss as a function of the pause time, based on cyclic voltammetry experiments with 1 M $NaPF_6$ dissolved in PC and EC:DEC (1:1 vol.) electrolytes using Solupor® (C-SP) and β-alumina (C-BA) separators, respectively. The pause order was long pause times before short pause times. With the Solupor® separator lower capacity losses were observed, implying that the species formed at the Na metal electrode resulted in a more stable SEI. With the β-alumina separator the SEI capacity loss in $NaPF_6$-PC was larger than in EC:DEC. The SEI formed in PC and EC:DEC shows different SEI stability depending on what separator was used: with the cell setup C-BA, the SEI in EC:DEC is more stable than in PC, whereas with C-SP cell setup, the SEI formed in PC is more stable over pause time. The cells have been tested once and therefore no error bars are shown. These results only serve the purpose to illustrate the qualitative difference between cell setup C-BA and C-SP.

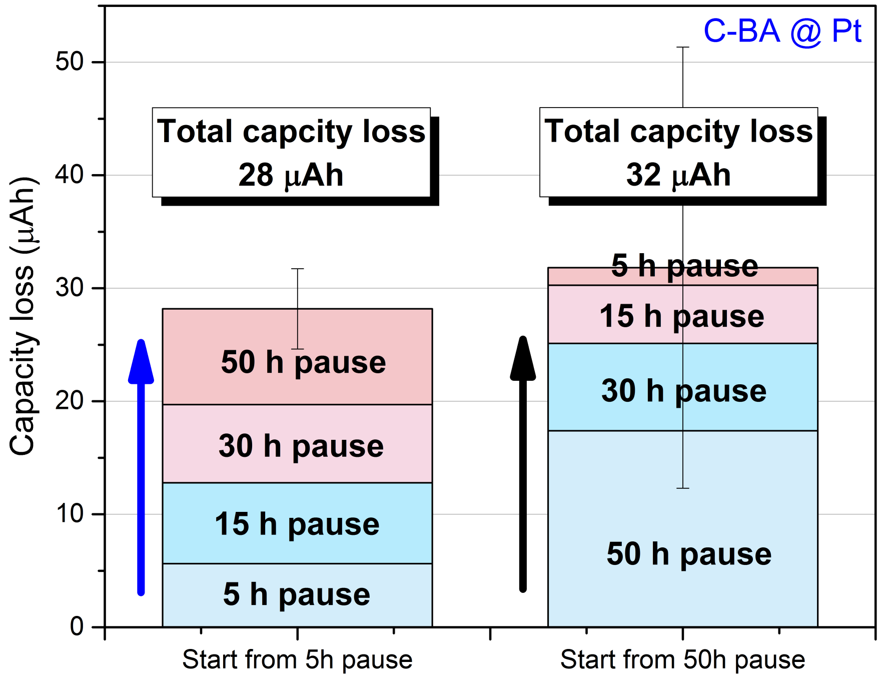

Figure S4. Sum of the average capacity losses after all pause times of 50 h, 30 h, 15 h and 5 h based on galvanostatic cycling results obtained in 1 M $NaPF_6$ EC:DEC electrolyte using the C-BA@Pt cell setup. In general, the total capacity loss is in both pause time orders (from short pauses to longer, shown with the blue arrow and from longer to shorter pauses, shown with the black arrow) ca. 30 µAh. Hence, the sum of capacity losses after the total amount of pauses does not depend on the pause order.

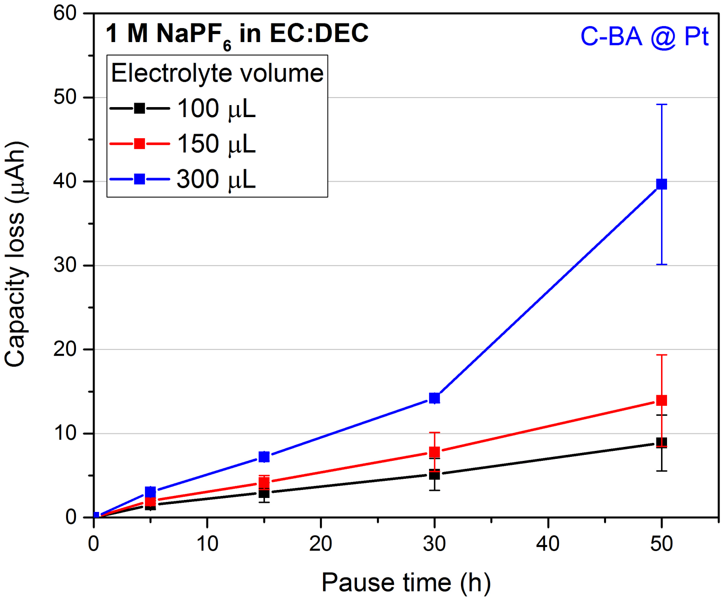

Figure S5. The capacity loss as a function of the pause time for different electrolyte (1 M $NaPF_6$ in EC:DEC) volumes using the C-BA @Pt cell setup.

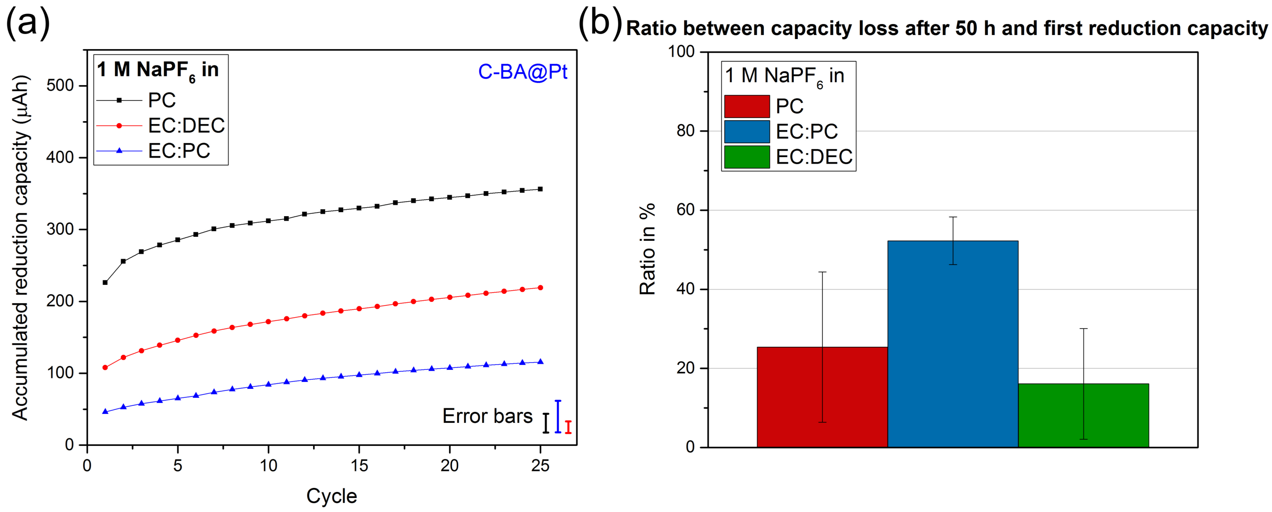

Figure S6. (a) Accumulated reduction capacity in 1 M $NaPF_6$ in PC (black), EC:DEC (red) and EC:PC (blue) as a function of the cycle number with error bars indicating the standard deviation based on the results from three cells. (b) The ratios between the capacity losses after 50 h and the first reduction capacity for the three different electrolytes. The capacity losses corresponding to 20-50 % of the initial reduction capacity indicates the presence of a SEI dissolution effect. The smallest capacity loss, due to SEI dissolution, was thus obtained with 1 M $NaPF_6$ dissolved in EC:DEC.

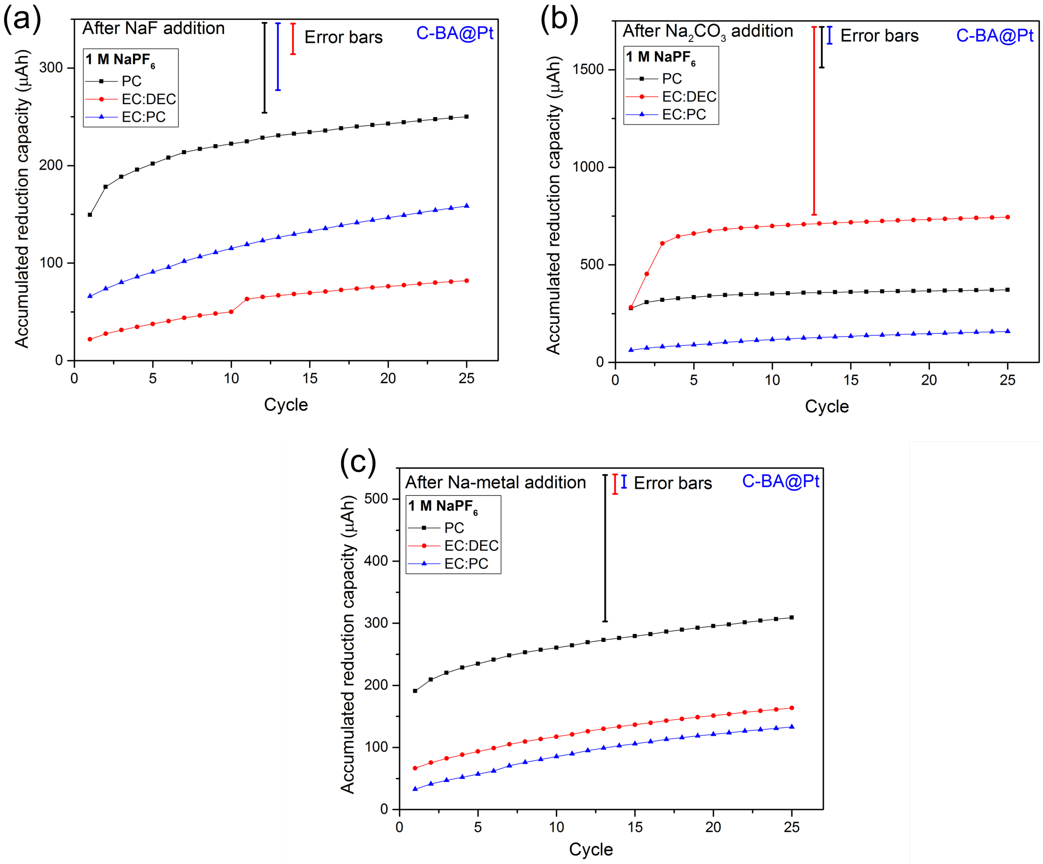

Figure S7. Accumulated reduction capacity in 1 M $NaPF_6$ in PC, EC:DEC and EC:PC after the addition of (a) NaF, (b) Na_2CO_3 and (c) Na metal as a function of the cycle number.

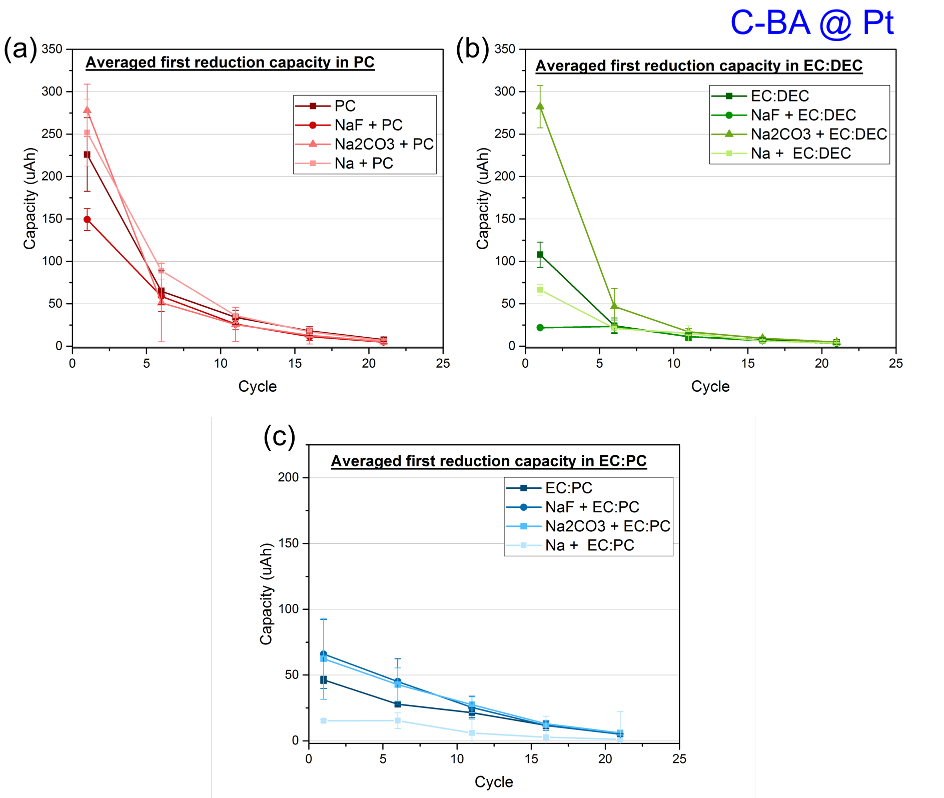

Figure S8. The results from the galvanostatic cycling presented in the main text (Figure 3, 4, 5 and 6) but presented for each different solvent to highlight the effect of the presence of the additives. For simplicity, only the first reduction capacities and subsequent capacities after a pause were plotted. The error bars represent the standard deviations based on the values obtained with three identical cells. The reduction capacity as a function of the cycle number for 1 M $NaPF_6$ dissolved in a) PC b) EC:DEC and c) EC:PC after addition of 10 mg/ml NaF and Na_2CO_3, and 60 mg/ml Na-metal, respectively. The additives were used to decrease the SEI dissolution rates, as mentioned in the main text. The testing with Na-metal serves the additional purpose of illustrating the effect of the presence of additional species formed at Na-metal electrode in the electrolyte when using the β-alumina separator. The $NaPF_6$-PC results were associated with significant uncertainties, and the effects of the additives were therefore not clear. The use of $NaPF_6$-EC:DEC and -EC:PC resulted in the lowest reduction capacities. For EC:DEC, the presence of NaF and Na-metal resulted in a lower reduction charge, whereas in EC:PC the presence of Na-metal decreased the first reduction charge. After 10 cycles, when the SEI formation was assumed to be essentially completed, the presence of additives did not have any significant effect on the results.

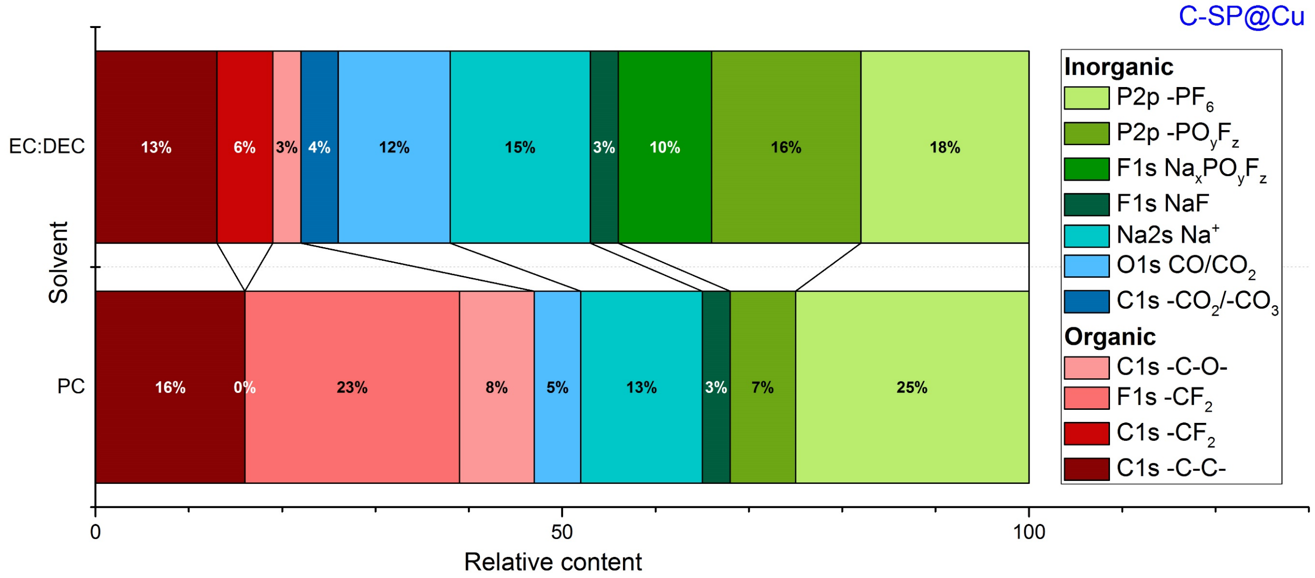

Figure S9. Relative contents of the different SEI species obtained from the peak integrals of the C 1s, O 1s, Na 2s, F 1s and P 2p spectra. The SEI layer formed in PC had a higher polymeric content than the SEI formed in EC:DEC which can be affected due to different water content in each electrolyte system. Compared to $NaPF_6$-EC:DEC, the SEI layer formed in the $NaPF_6$-PC electrolyte featured higher relative amounts of C-C, C-O, $-CF_2$ species indicating more polymer formation. The SEI layer obtained in the $NaPF_6$-EC:DEC electrolyte was, on the other hand, more inorganic due to the presence of more carbonate containing compounds. The phosphorus and fluorine containing SEI components were similar in PC and EC:DEC which could be explained by products stemming from the $NaPF_6$ salt, such as POF_3 and PF_5^-. It should, however, be pointed out that the SEI composition could have been affected by species formed at the Na metal anode since the experiments were carried out with a cell containing a Solupor® separator. The results should therefore be carefully evaluated with the electrochemical and ICP-OES data in mind.

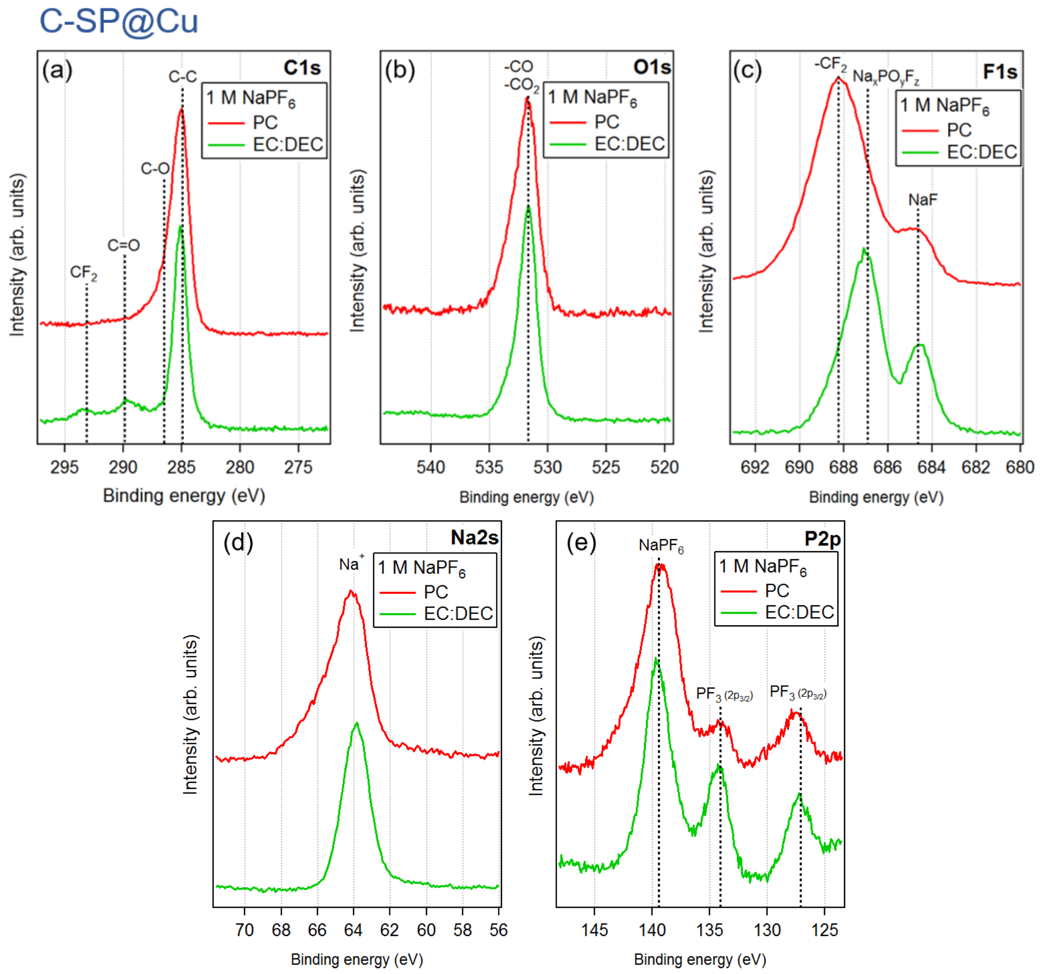

Figure S10. XPS-spectra of the SEI layers formed after the cycling of a Cu-foil between 0.1 and 2.0 V vs. Na^+/Na and stopping the scan at 2.0 V after 10 cycles. (a) C 1s, (b) O 1s, (c) F 1s, (d) Na 2s and (e) P 2p. The XPS spectra indicate that there was no significant influence of the conversion reaction involving the native copper oxide layer on the Cu-foil since there was no Na_2O peak (529.70 eV) in the O 1s spectra.

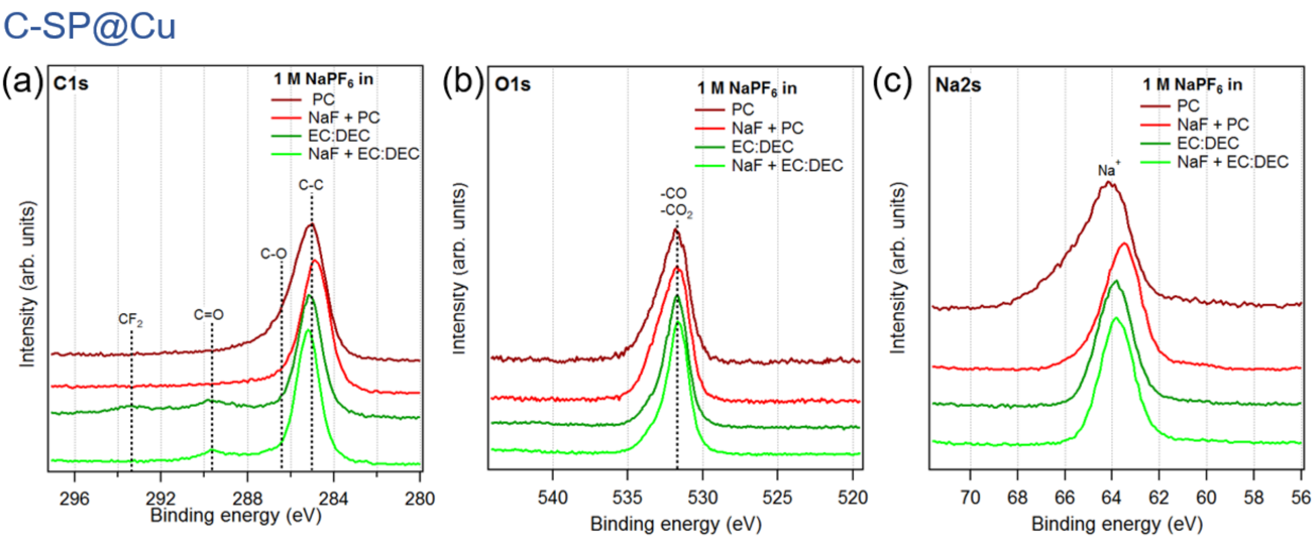

Figure S11. XPS-spectra of the SEI layer formed after the cycling of a Cu-foil between 0.1 and 2.0 V vs. Na^+/Na and stopping the scan at 2.0 V after 10 cycles. (a) C 1s, (b) O 1s, (c) Na 2s before and after NaF addition (10 mg/ml). The Na 2s spectra show a spectral change, before and after NaF addition for $NaPF_6$-PC electrolyte. After the NaF addition, the Na 2s core level was similar to that seen the $NaPF_6$-EC:DEC electrolyte.

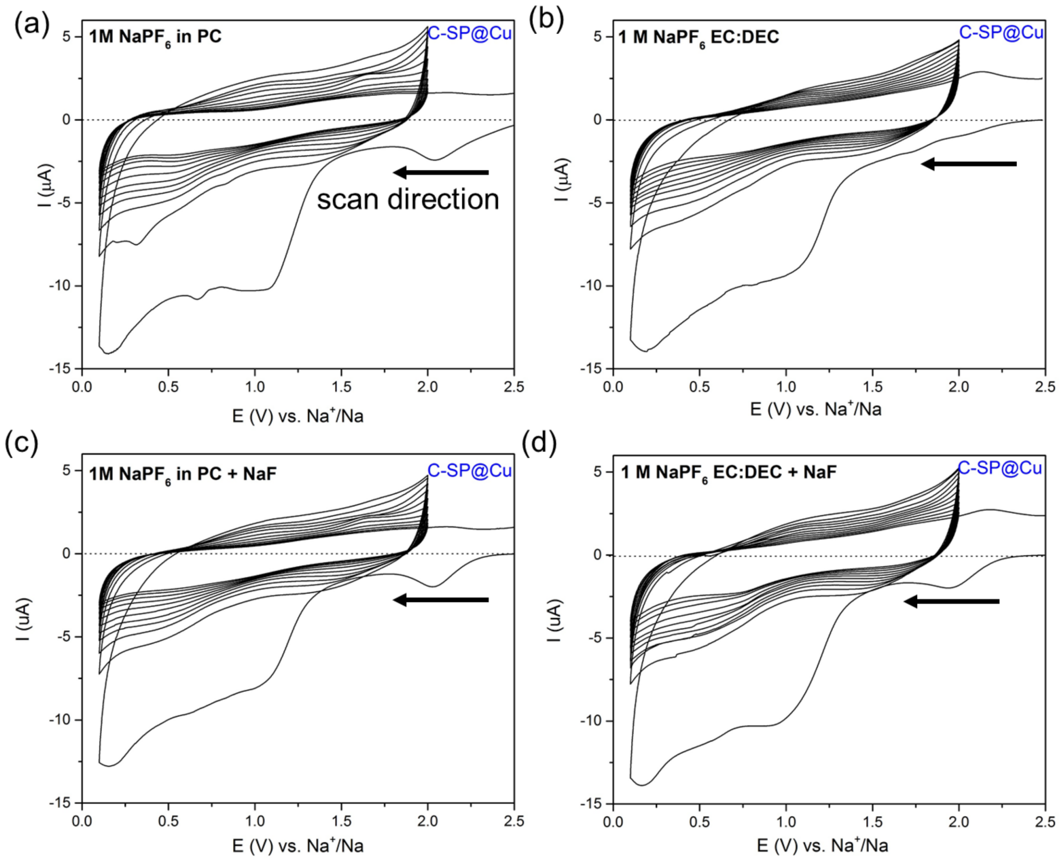

Figure S12. Cyclic voltammograms recorded with the C-SP@Cu cell setup prior to the for XPS measurements used to illustrate the influence of the NaF additions. The cells were cycled from 0.1 to 2.0 V vs. Na^+/Na at a scan rate of 0.5 mV/s in (a) 1 M $NaPF_6$ in PC, (b) 1 M $NaPF_6$ in EC:DEC, (c) 1 M $NaPF_6$ in PC + NaF (10 mg/ml) and (d) 1 M $NaPF_6$ in EC:DEC + NaF (10 mg/ml). The scan direction is shown in the voltammograms.

Table S1. First reduction capacities and the associated errors expressed as ± one standard deviation based on three samples.

Electrolyte solvent	Control [µAh]		NaF [µAh]		Na_2CO_3 [µAh]		Na-metal [µAh]	
	Value	*Error ±*	*Value*	*Error ±*	*Value*	*Error ±*	*Value*	*Error ±*
**PC**	226	43	149	13	278	31	252	39
**EC:PC**	46	3	66	26	62	31	39	15
**EC:DEC**	108	15	22	0.1	282	25	67	6

Table S2. Self-discharge rates and the associated errors expressed as ± one standard deviation.

Electrolyte solvent	Control [µAh]		NaF [µAh]		Na_2CO_3 [µAh]		Na-metal [µAh]	
	Value	*Error ±*	*Value*	*Error ±*	*Value*	*Error ±*	*Value*	*Error ±*
**PC**	1.135	0.018	0.872	0.095	0.887	0.020	1.494	0.130
**EC:PC**	0.494	0.022	0.790	0.020	0.757	0.009	0.809	0.010
**EC:DEC**	0.335	0.017	0.393	0.018	0.639	0.027	0.355	0.018

Author Contributions

L.A.M. and R.Y. designed the experiments. L.A.M. performed the ICP-OES and electrochemical measurements. A.J.N conducted the XPS measurements and L.A.M analysed the data. L.A.M., L. N. and R.Y. participated in the discussion of the electrochemical data. L.A.M. analysed the data and wrote the manuscript with the help of all co-authors. R.Y. led the project.

References

[1] R. Mogensen, D. Brandell, R. Younesi, *ACS Energy Lett.* **2016**, *1*, 1173–1178.
